# Genome wide identification and predicted functional analyses of NAC transcription factors in Asian pears

**DOI:** 10.1186/s12870-018-1427-x

**Published:** 2018-10-03

**Authors:** Mudassar Ahmad, Xinhui Yan, Jianzhao Li, Qinsong Yang, Wajeeha Jamil, Yuanwen Teng, Songling Bai

**Affiliations:** 1 Department of Horticulture, Zhejiang University, Hangzhou, 310058 Zhejiang China; 2 The Key Laboratory of Horticultural Plant Growth, Development and Quality Improvement, the Ministry of Agriculture of China, Hangzhou, 310058 Zhejiang China; 3 Zhejiang Provincial Key Laboratory of Integrative Biology of Horticultural Plants, Zhejiang, 310058 Hangzhou China

**Keywords:** Pear, Genome-wide analysis, NAC transcription factors, *PpNAC* predicted functions, Bud endodormancy, Fruit color, Fruit development

## Abstract

**Background:**

NAC proteins contribute to diverse plant developmental processes as well as tolerances to biotic and abiotic stresses. The pear genome had been decoded and provided the basis for the genome-wide analysis to find the evolution, duplication, gene structures and predicted functions of *PpNAC* transcription factors.

**Results:**

A total of 185 *PpNAC* genes were found in pear, of which 148 were mapped on chromosomes while 37 were on unanchored scaffolds. Phylogeny split the NAC genes into 6 clades (Group1- Group6) with their sub clades (~ subgroup A to subgroup H) and each group displayed common motifs with no/minor change. The numbers of exons in each group varied from 1 to 12 with an average of 3 while 44 pairs from all groups showed their duplication events. qPCR and RNA-Seq data analyses in different pear cultivars/species revealed some predicted functions of *PpNAC* genes i.e. *PpNACs 37, 61, 70* (2A), *53, 151*(2D), *10, 92, 130* and *154* (3D) were potentially involved in bud endodormancy, *PpNACs 61, 70* (2A), *172, 176* and *23* (4E) were associated with fruit pigmentations in blue light, *PpNAC*s *127* (1E), *46* (1G) and *56* (5A) might be related to early, middle and late fruit developments respectively. Besides, all genes from subgroups 2D and 3D were found to be related with abiotic stress (cold, salt and drought) tolerances by targeting the stress responsive genes in pear.

**Conclusions:**

The present genome-wide analysis provided valuable information for understanding the classification, motif and gene structure, evolution and predicted functions of NAC gene family in pear as well as in higher plants. NAC TFs play diverse and multifunctional roles in biotic and abiotic stresses, growth and development and fruit ripening and pigmentation through multiple pathways in pear.

**Electronic supplementary material:**

The online version of this article (10.1186/s12870-018-1427-x) contains supplementary material, which is available to authorized users.

## Background

Transcription factors are master regulators which mediate transcriptional regulations by binding a specific nucleotide sequence in response to developmental and environmental changes in plants. As a crucial type of TFs, NAC, a term derived from 3 genes i.e. NAM (No apical meristem), ATAF 1/2 (Arabidopsis transcription activator factor 1/2) and CUC2 (cup shaped cotyledon) [1], is not only one of the largest transcription factors, but also has an important position in plant developments and stress regulations [2]. Protein sequences of this family indicate that the N-terminal region (NAC domain) of these proteins is highly conserved and comprises about 160 amino acid residues and involve in DNA binding while the C-terminal region is highly diversified in length and sequence and consider as the transcriptional activation domain [3]. The N-terminal region (NAC domain) is further divided into 5 subdomains (A-E) of conserved blocks embedded in heterogeneous blocks or gaps. “A” subdomain promotes functional dimerization, “B” and “E” has distinctive functions of protein while “C” and “D” are positively charged and allow the TF to bind to the DNA [4–7].

More than 100 members of NAC TFs are extensively distributed in the plant genome while a few of them are found to have diverse biological processes, including formation and maintenance of shoot apical meristem [8], embryo, seed and lateral root developments [9–11], hormone signaling [12], cell division [13], regulation of secondary cell wall synthesis [14] and fruit ripening, growth and coloration [15, 16]. In particular, NAC TFs have got much attention as regulators in plant tolerance pathways during both biotic and abiotic stresses. For instance, *AtAF1* and *AtAF2* related to fungal diseases in Arabidopsis [17], *OsNAC6* enhanced high salinity, dehydration and disease tolerances in transgenic rice plants [18], *StNAC* gene was related to pathogen attack and wounding in potato [19], drought and cold induced *BnNAC* genes in Brassica [20], *SNAC1/2* helped in drought resistance and salt tolerance in rice [21, 22] and overexpression of *AtNAC019, AtNAC072*, and *AtNAC055* increased drought and stress tolerances [6] while *AtNAC2* enhanced salt tolerance through ethylene signaling pathway in Arabidopsis [23]. These biotic and abiotic stresses are the major factors that cause limiting growth and productivity [24] and NAC TFs are up and down regulated during these limitations in both herbaceous as well as woody plants.

Pear from Rosaceae family is one of the most important temperate deciduous perennial woody plants. The NAC genes may play fundamental roles on the development and biotic/abiotic stresses in pear and other rosaceous plants as well. As in pear, *PbeNAC1* was involved in cold and drought tolerances via modulating the expression of stress-responsive genes (*NtRD29A*, *NtRD17* etc), biosynthesis of proline (*NtP5CS*) and ROS scavenging enzymes (*NtCAT*, *NtSOD*, and *NtAPX*) in transgenic tobacco through interacting with dormancy related *PbeDREB1* and *PbeDREB2A* genes [25]. In apple, *MdNAC029* negatively regulated cold tolerance via CBF-dependent pathway [26] while *MdNAC047* enhanced the salt stress tolerance by modulating the ethylene response [27]. NAC TF was also be found to be highly overexpressed in blood-fleshed peaches as compared to non-red-fleshed peaches and *PpeNAC1* helped in anthocyanin accumulation in tobacco by interacting with *PpeMYB10* [16].

To date, genome wide analyses of NAC TFs have been investigated in various plant species such as Arabidopsis [3], rice [28], grapes [29], tobacco [30], soybean [31], apple [32], cabbage and wheat [33]. We performed the genome wide and predicted functional analyses of *PpNAC* TFs based on the draft genome sequence of pear [34]. Given the importance of this family, the chromosome location, gene structures and protein motifs of the putative *PpNAC* genes predicted by genome wide surveys were carefully analyzed. The putative pear *NAC* genes were further subjected to phylogenetic analyses with Arabidopsis and apple. These comparisons enabled the identification of genes for their further characterizations. Moreover, we paid additional attentions to find the predicted *PpNAC* genes related to stress tolerance, bud endodormancy, fruit pigmentation and fruit development.

## Results

### Identification of *NAC* gene family in pear

The first strategy of Hidden Markov Model search (HMM search) with the HMM profile (PF01849) of the NAM domain against the pear predicted protein database and the other strategy of plant transcription factor database resulted in 230 *PpNAC* proteins. The sequences which were redundant, overlapped, incomplete and repeated were rejected and further manually analyzed using Interproscan and SMART blast to reconfirm the presence of the NAM domains in each sequence. Finally, 185 sequences containing typical NAM domain with full ORFs were identified and considered as the pear NAC transcription factors. The detailed information and protein sequences of selected pear NAC TFs is listed in Additional file 1.

#### Phylogenetic analysis of *PpNAC* TFs

**Fig. 1:**
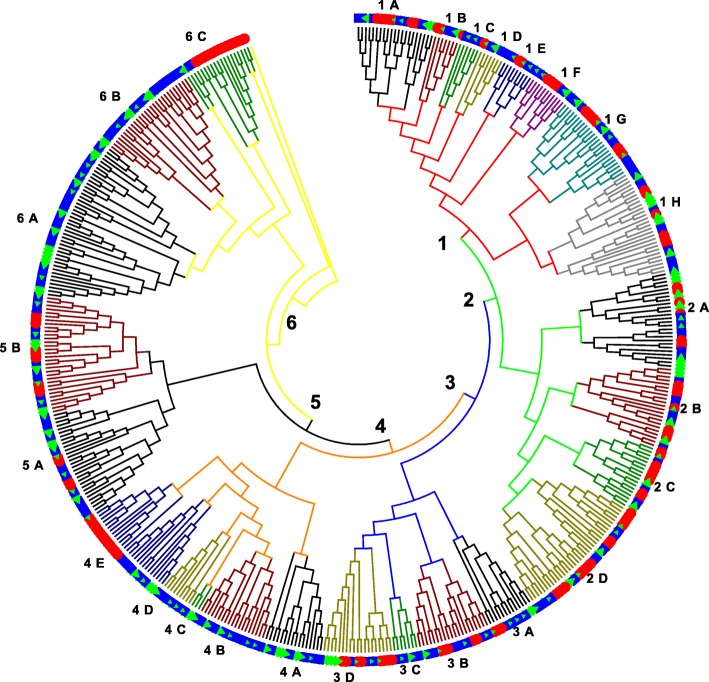
Phylogenetic analysis of the NAC transcription factors of Arabidopsis, apple and pear represented by red circles, blue squares and green triangles respectively. The full-length sequences of the NAC proteins were aligned using MUSCLE, and the phylogenetic tree was made using the maximum-likelihood tree method in the MEGA7.0 software. The tree was divided into 6 main groups 1, 2, 3, 4, 5 and 6 represented by red, green, blue, orange, black and yellow colored clusters within the tree while their subgroups designated from 1A-1H, 2A-2D, 3A-3D, 4A-4E, 5A-5B and 6A-6C respectively outside the tree

To explore the phylogenetic relationships among 185 *PpNAC* genes and to clarify the evolutionary relations within this family, a combined phylogenetic tree of pear was constructed with apple and Arabidopsis NAC proteins. Due to high diversity in sequence lengths, a maximum likelihood phylogenetic tree was constructed which split the NAC proteins into 6 distinct clades (Group1- Group6) with their sub clades (~ subgroup A to subgroup H) (Fig. 1). Group 1 was the largest one with 40 *PpNAC* genes and 8 subgroups (1A-1H) followed by groups 2 and 6 with 34 genes of each and 4 (2A-2D) and 3 (6A-6C) subgroups respectively (Table 1). Further, members of *PpNAC* TFs in groups 1, 2, 3 and 5 were clustered with the members of apple and Arabidopsis although some species-specific subgroups were also been identified (subgroup 3A). In contrary, genes of pear and apple in groups 4 and 6 were separately clustered with those of Arabidopsis and demonstrated some independent evolutionary history between Arabidopsis and Rosacea plants. Detailed phylogenetic tree of apple, pear and Arabidopsis is in Additional file 2.

**Table 1:** Group wise distributions and predicted functions of *PpNAC* transcription factors

GROUP#	Subgroups	No. of genes	Exons #	Domain#	Best Smart hit	Submitter description	Reference
GROUP1	1A	7	3	1,4,2,5,3,6,7	NAC100	Cell expansions	[40]
	1B	3		1,2,5,3,6,7	CUC2	Regeneration in root explants	[60]
	1C	2		1,4,2,5,3,6,7	CUC3	Anthocyanin biosynthesis in peach	[16]
	1D	3		1,4,2,5,7	CUC1		
	1E	3		1,4,2,5,3,6,7	CUC2 LIKE		
	1F	4		1,4,2,5,3,6,7	NAC21/22		
	1G	7		1,4,2,5,3,6,7	BEARSKIN2 LIKE	Root cap maturation	[49]
	1H	11		1,4,2,5,3	VND	Vascular and secondary cell wall formation	[50]
	Subtotal	40					
GROUP2	2A	13	3	1,4,2,5,3,6,7	JUNGBRUNNEN 1-like isoform	GA and BR signaling	[41]
	2B	8	6	1,4,2,5,3,6,7	NAC28		
	2C	5	5	1,4,2,5,3,6,7	NTM1, TCV	Salt stress response	[42]
	2D	8	5	1,4,2,5,3,6,7	NTL 8	Seed germination during salt stress	[42]
	Subtotal	34					
GROUP3	3A	6	3	1,4,2,5,3,6,7	NAC83		
	3B	6	3	1,4,2,5,3,6,7	NAC2	Stamen development in arabidopsis	[61]
	3C	3	2	1,4,2,5,3,6,7	NARS1	Embryogenesis	[62]
	3D	8	3	1,4,2,5,3,6	AtAF1	Salt stress and ABA signaling in rice	[63]
	Subtotal	23					
GROUP4	4A	10	3	1,5,3,7	NAC67	Group4 genes in *PpNAC* and *MdNAC* TFs showed independent evolution with *AtNAC* TFs. Anthocyanin accumulation in pear (Present Study)	
	4B	7	6	1,4,2,5,3,6	NTM1 like		
	4C	3	6	1,4,2,5,3,7	NAC isoform X1		
	4D	3	6	1,4,2,5,3,6,7	NAC83 like		
	4E	7	4	1,4,5,3,6,7	NAC14		
	Subtotal	30					
GROUP5	5A	10	3	1,4,5,3,6,7	AtNAP	Fruit and Leaf senescence	[64]
	5B	14	3 & 8	1,2,6,7	AtVNI2	Abscisic acid signaling	[65]
	Subtotal	24					
GROUP6	6A	22	1, NO INTRON	1,8,9,10,6,7	NAC41/CUC1 like	Group6 genes in *PpNAC* and *MdNAC* TFs showed independent evolution with *AtNAC* TFs	
	6B	10	2	1,8,2,6,7	NAC30		
	6C	2	3	1,7	NAC30 like		
	Subtotal	34					
TOTAL		185					

Previous studies on NAC TFs demonstrated that genes clustered in one subgroup might have tendency to do similar functions. So, we searched some predicted functions of *PpNAC* groups based on the published papers. Our findings revealed that group 1 might belong to cell growth and development while group 2, 3 and 5 performed some signaling roles during biotic and abiotic stresses. Although there were some specificity in predicted functions at subgroup and gene levels in *PpNAC* TFs as shown in Table 1. But, functional evaluations of NACs from groups 4 and 6 were hard to be predicted due to independent evolution (Fig. 1 & Table 1).

### Genome distribution and duplication analysis of *PpNAC* gene family

**Fig. 2:**
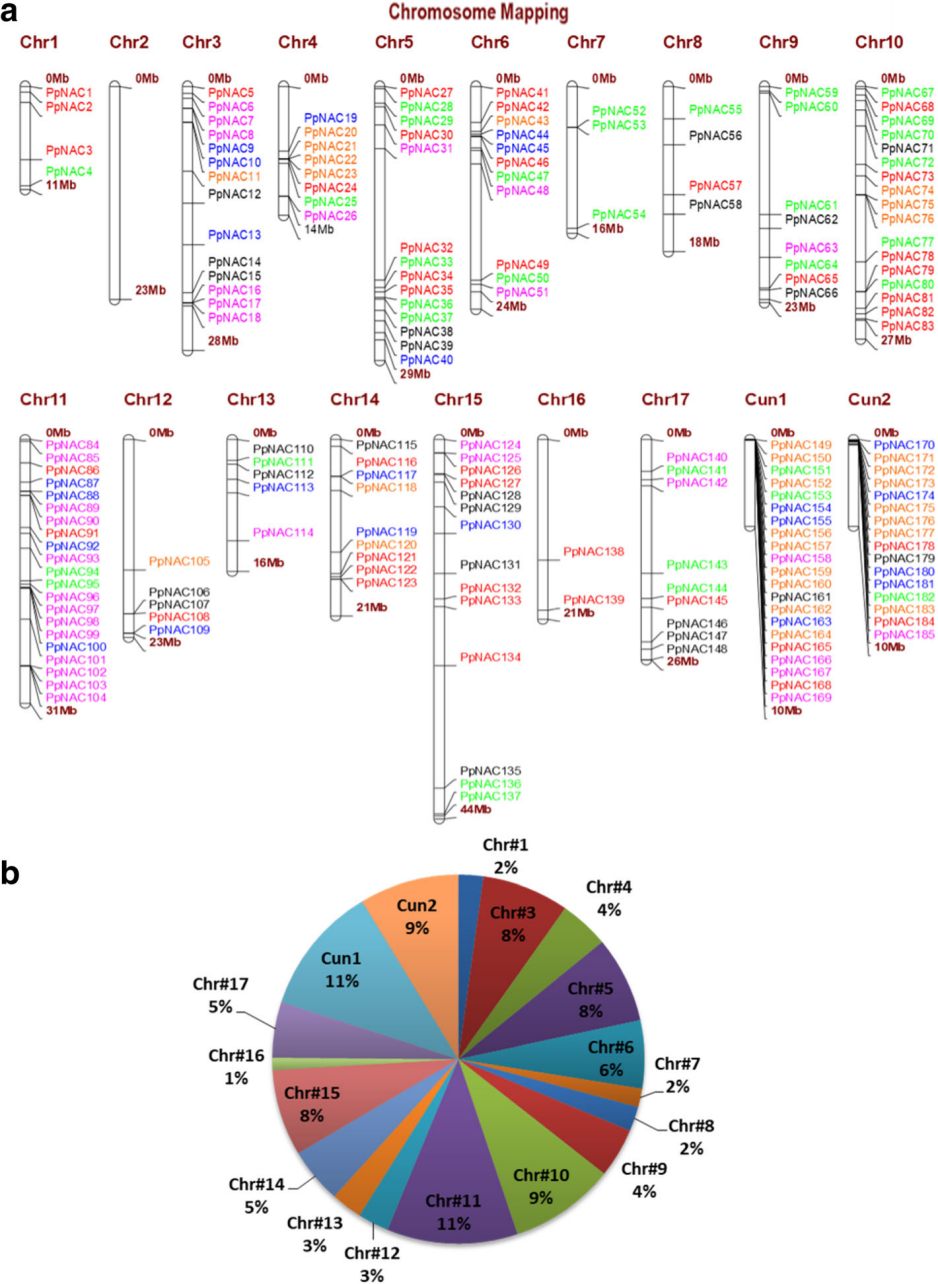
Chromosomal mapping and frequency distribution of the *PpNAC* transcription factors. **a**, Physical locations of the putative *PpNAC* genes on each chromosome. Red, green, blue, orange, black and purple indicate groups 1, 2, 3, 4, 5 and 6 respectively. The chromosomal length is shown at the end while the chromosome number (Chr01-Chr17) is shining at the top. *PpNAC* genes located on scaffold are mapped on Cun1 and Cun2 with 10 Mb lengths. **b**, Number of *PpNAC* genes according to their frequency distributions on each chromosome. Each color shows different chromosome numbers

The chromosome locations of all *PpNAC* genes were identified using BLAST software 2.25 to align these *PpNAC* sequences against the genome sequences of pear. The physical map positions demonstrated that in total, 148 (81%) *PpNAC* genes were localized on 16 chromosomes in ascending order from short arm telomere to long arm telomere and interestingly chromosome 2 was unoccupied by *PpNAC* genes (Fig. 2a). The remaining 37 genes could not be conclusively charted on chromosomes due to presence on unanchored scaffolds, so they were mapped on Cun1 (11%) and Cun2 (8%) in ascending order. The given names of *PpNAC* TFs were according to these ascending order arrangements in chromosomes as well as Cuns. The position and length of each gene are shown in Additional file 1. Maximum *PpNAC* gene frequencies were observed on chromosome 11 (11%) followed by chromosome 10 (9%) while minimum frequencies were shown by chromosome 16 and 7 (1% and 1.6%) (Fig. 2b; Additional file 3). The maximum percentage of groups 1, 2, 3, 4, 5 and 6 were located on chromosome 10 (17.5%), chromosome 10 (17.6%), chromosome 11 (17%), chromosome 4 (13%), chromosome 15 (16%) and chromosome 11 (38%) respectively while group 4 were also highly mapped on Cun1 (33%) and Cun2 (23%) (Additional file 3).

**Fig. 3:**
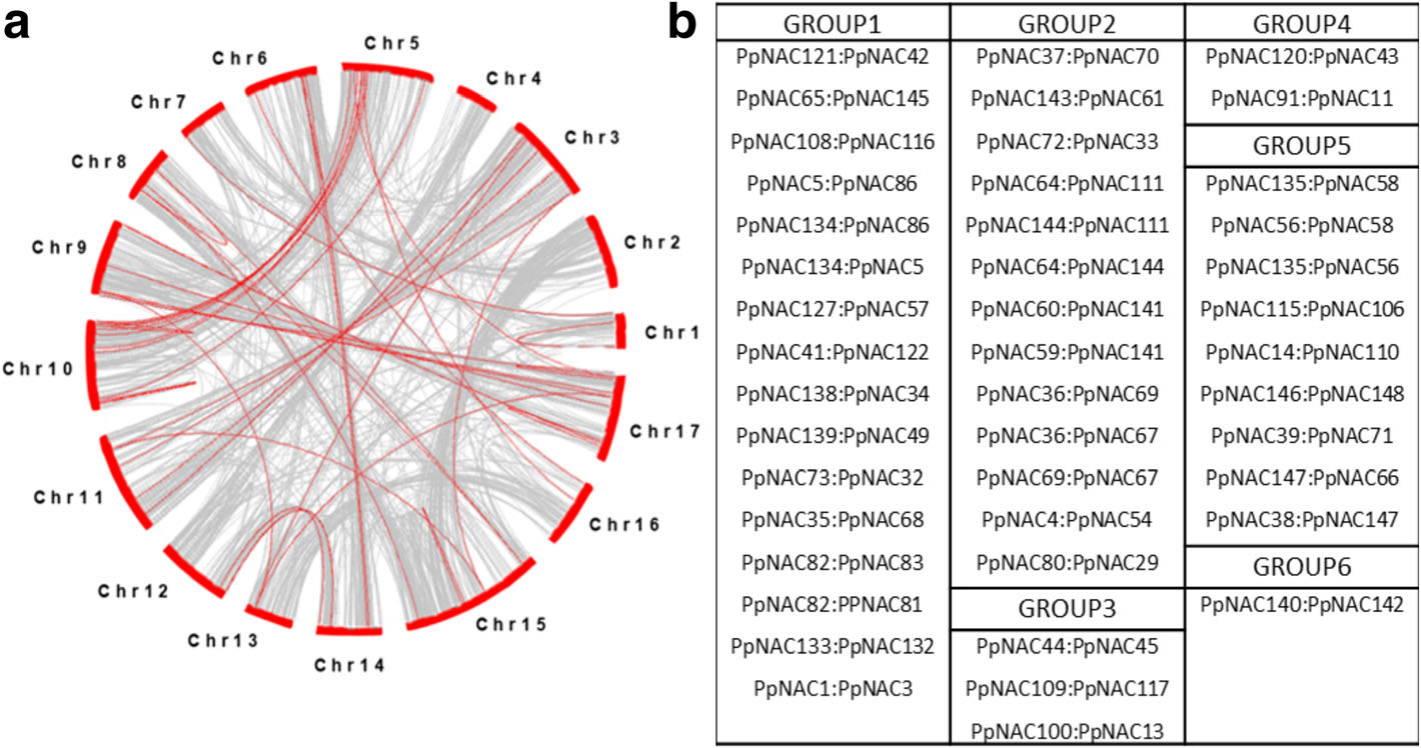
Collinearity analysis of *PpNAC* genes. **a**, Red lines indicate the *PpNAC* genes duplication while the gray lines indicate the whole genome duplication events. Red bars indicate each chromosome number. **b**, Duplicated gene pairs of *PpNAC* TFs according to their assigned groups

Due to the presence of a whole-genome duplication event in the evolution of pear, gene duplication events within pear NAC family had also been detected as similar in several plants. To find out the duplication events in *PpNAC* TFs, a collinerity analysis had been performed by using MCScanX software. In 185 *PpNAC* TFs, we found 44 pairs of duplicated genes (Fig. 3a, b). Maximum duplication events were seen in group 1 followed by group 2 while groups 4 and 6 showed very less duplication events (Fig. 3b).

### Gene structure and protein motifs of *PpNAC* TFs

**Fig. 4:**
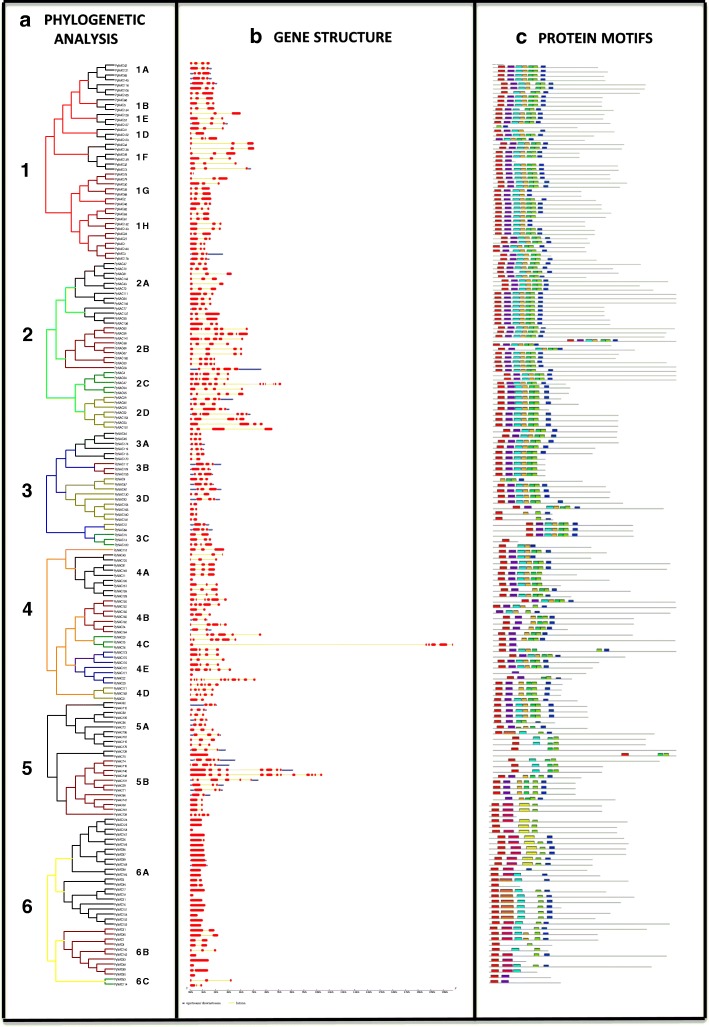
Phylogenetic tree of all 6 groups of *PpNAC* transcription factors, their gene structures and protein motifs: **a**, Phylogenetic tree: The amino acid sequences of all 6 groups were aligned using MUSCLE, and the phylogenetic tree was constructed using the neighbor-joining method through MEGA7 software. Groups are shown by numbers from 1 to 6 with red, green, blue, orange, black and yellow color trees while their subgroups are showed with alphabet letters. **b**, Gene structure: The exons are represented by red boxes while introns with yellow lines and blue bars indicate the upstream or downstream which was elucidated by using Gene Structure Display Server. The scale bar is represented 1.0 kb (middle). **c**, Protein motif: schematic diagrams of possible conserved motifs in PpNAC proteins, MEME tool was used to find out the conserved motifs. Conserved motifs (1–10) are indicated by different colored boxes while the non-conserved sequences are showed by the gray lines

Among the *NAC* family, the closest members in the same subgroups shared a similar exon/intron structures in terms of intron numbers and exon lengths (Fig. 4a, b). For instance, group A with all its subgroups had 3 exonic and 2 intronic regions and subgroup 6 had no or one intronic region and seemed to be the smallest in structure than all others. Group 5 possessed the biggest structure and most numbers of exon and intron in their members (Fig. 4b). In contrast, members of subgroups 2B, 2C, 4C, 4E, and 5B showed a large variability in either the distribution or number of introns (Table 1). If specifically highlighted, *PpNAC 148* (5B) had the biggest gene structure with 12 intronic parts followed by *PpNAC 23* (4E) and *PpNAC 47* (2C) while *PpNAC 76* (4C) had the biggest intron structure than others (Fig. 4b). In short, the coding regions in *PpNAC* TFs varied from 1 to 12 with an average of 3 as found in NAC population of other species. These gene structures also provided the reliable substantiation to support and authenticate our phylogenetic groupings and subgroupings in *PpNAC* genes.

To further explore the diversification and potential conserved motifs in *PpNAC* genes, putative motifs were predicted by the Multiple Em for Motif Elicitation (MEME) tool and 10 divergent motifs were recognized in *PpNAC* TFs and named as motifs 1–10 (Fig. 4c). Motif 1 is recognized as NAM while others (Motifs 2–10) were remained unrecognized. As expected, we found that most of the groups based on the phylogeny displayed common motifs with same alignment and position while there were no/minor differences at subgroup levels. So, NAC proteins with similar gene structures and motifs were present in the same subgroups and might have the similar functions as shown in Table 1.

### Expression analysis of *PpNAC* genes based on RNA-Seq datasets

To find out the possible roles of *PpNAC* genes, their expression profiles were analyzed based on the three published RNA-Seq datasets: (i) pear bud dormancy in *Pyrus pyrifolia* ‘Suli’ [35]; (ii) fruit color development in *P. pyrifolia* ‘Meirensu’ [36] and (iii) different fruit development stages in *P. pashia, P. calleryana*, and *P. pyrifolia* [37]. The expression patterns of *PpNAC* genes were classified into three types based on their expression profiles i.e. constitutive expressions, period specific expressions and no expressions.

**Fig. 5:**
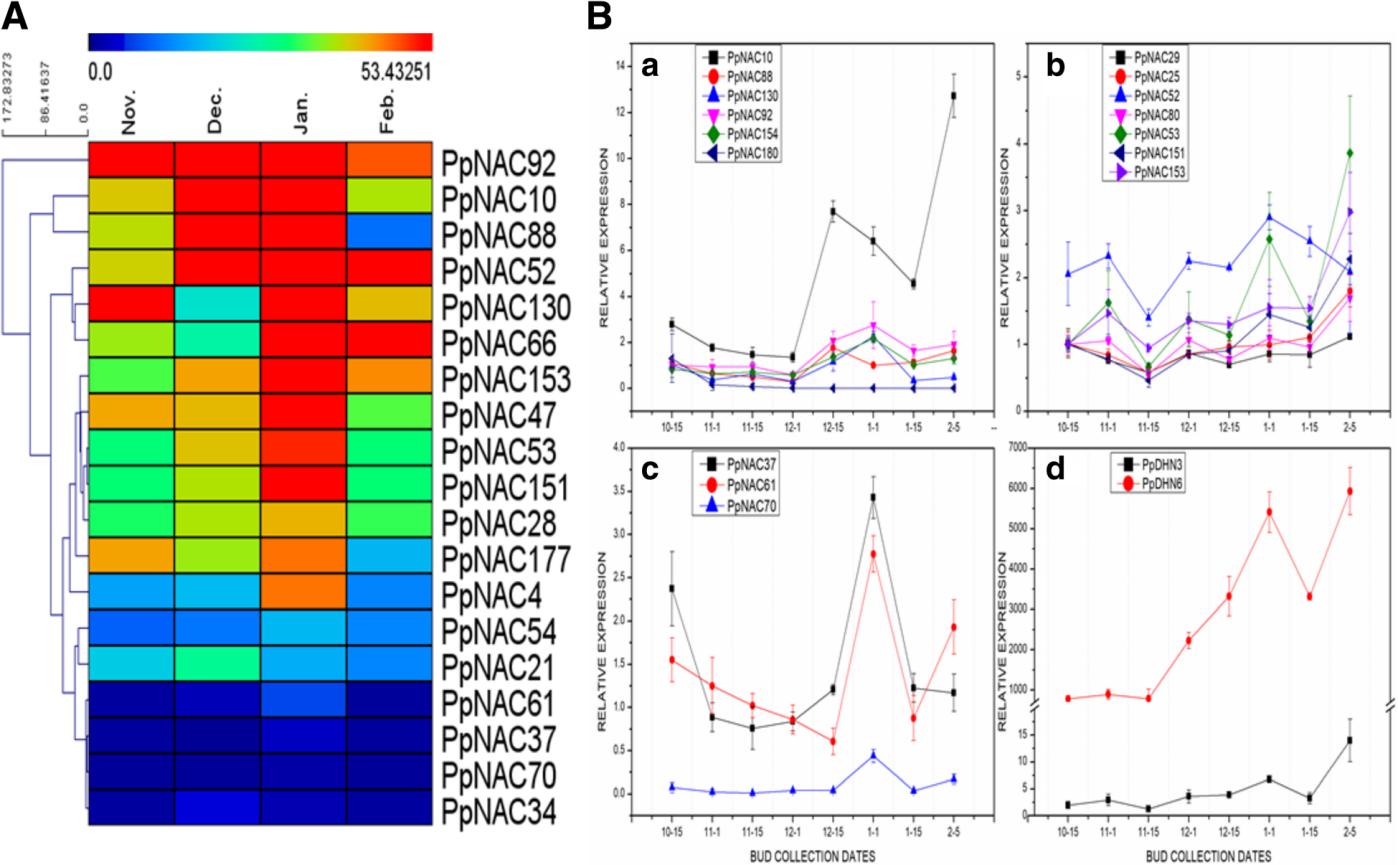
Heat map presentation and expression analysis of selected *PpNAC* TFs during bud endodormancy in different pear cultivars. **A**, Heat map from RNA-seq data of differentially expressed *PpNAC* TFs during bud endodormancy period in *P. pyrifolia* ‘Suli’ during the entire endodormancy cycle. The color key at the top of heat map represents log2 of RPKM values. **B**, Expression analysis of the identified *PpNAC* genes during bud endodormancy in *P. pyrifolia* cv. Cuiguan. **B**-a, b, c, The expression patterns of selected genes from subgroups 3D, 2D and 2A in pear buds collected from Oct.-15 to Feb.-5 during the endodormancy period respectively. **B**-d, Endorsement of dormancy is assessed by the expressions of dehydrin genes with same samples. The data were normalized to the pear *ACTIN* expression level and the mean expression value was premeditated from 4 independent replicates while the standard deviation are also shown by vertical bars

**Fig. 6:**
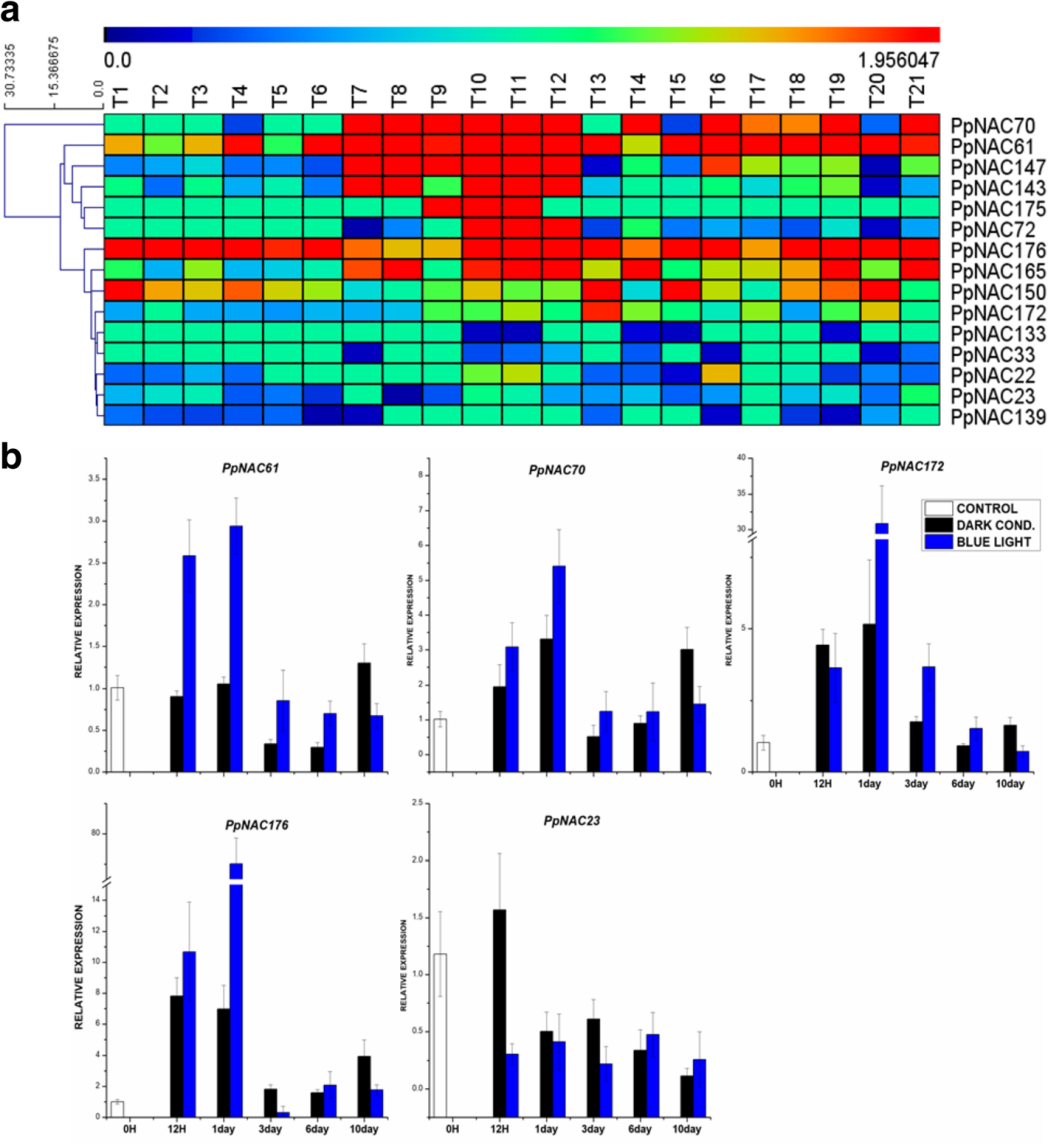
Heat map presentation and expression analysis of selected *PpNAC* TFs during fruit color development in different pear cultivars. **a**, Heat map of *PpNAC* TFs during fruit color development under sun light and dark light treatments i.e. T1-T3, T4-T6, T7-T9 and T10-T12 indicates 0 h, 6 h, 1 day and 6 days under sun light conditions while T13-T15, T16-T18 and T19 to T21 represents 6 h, 1 day and 6 days treatments under dark conditions respectively with three independent biological replicates of *Pyrus pyrifolia* ‘Meirensu’. The color key at the top of heat map represents log2 of RPKM values. **b**, Expression analysis of the identified *PpNAC* genes during fruit color development in *P. pyrifolia* ‘Red Zaosu’. Expression representation of some genes from subgroup 2A (*PpNAC61, PpNAC70*) and 4E (*PpNAC172, PpNAC176, PpNAC23*) under blue light and dark treatments with 0 h, 12 h, 1 day, 3 days, 6 days and 10 days interval. The data were normalized to the pear *ACTIN* expression level and the mean expression value was premeditated from 4 independent replicates while the standard deviation are also shown by vertical bars

**Fig. 7:**
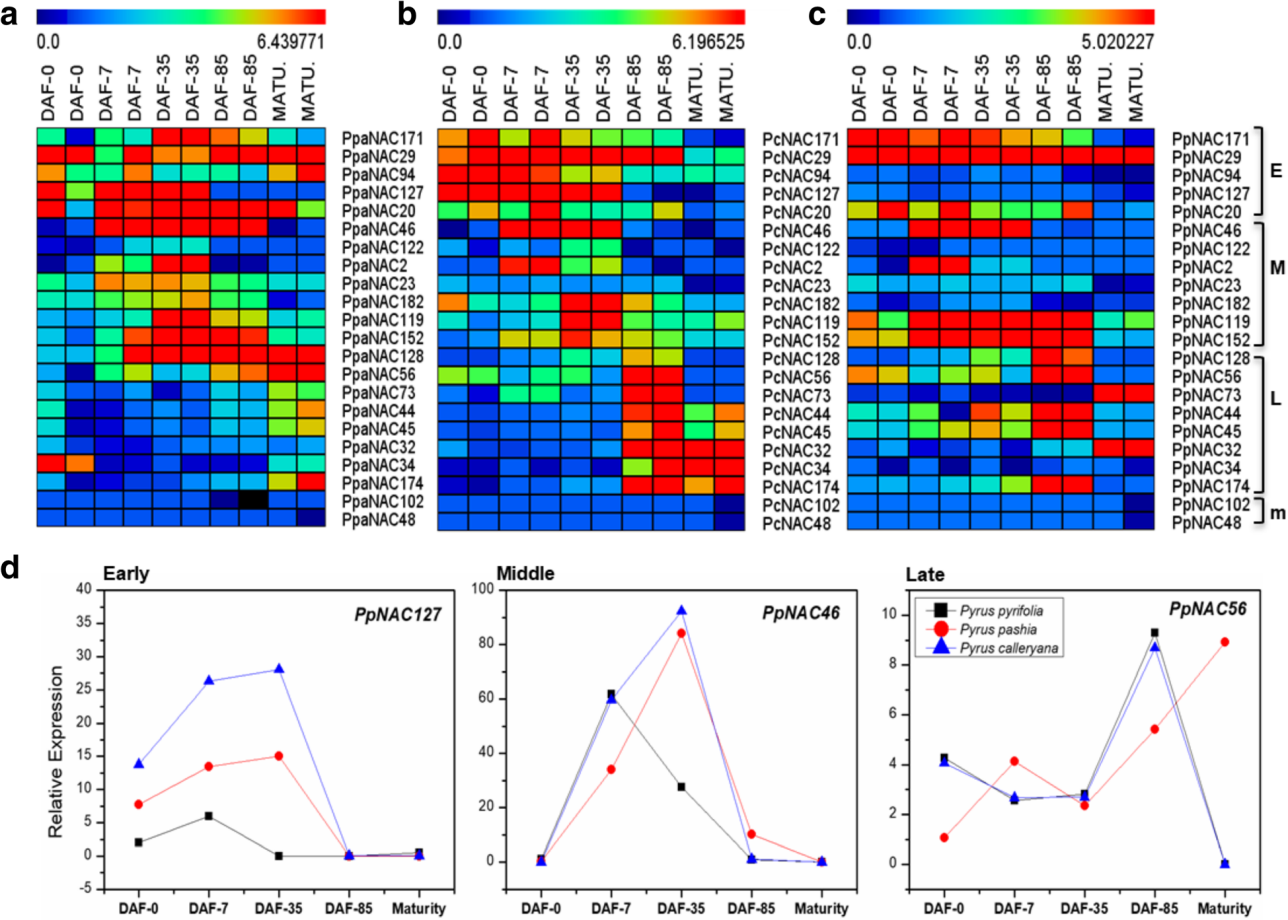
Heat map and graphical presentations of selected *PpNAC* TFs during fruit development in three different pear cultivars. **a**, **b**, **c** Expression profiles of *PpNAC* TFs from 0, 7, 35 and 85 days after flowering to fruit maturity with two replicates in *P. pashia, P. calleryana*, and *P. pyrifolia* respectively. E, M, L and m indicates early, middle, late and maturity related *PpNAC* genes respectively. The color key at the top of heat map represents log_2_ of RPKM values. **d**, Graphical representations of *PpNAC* genes related to early, middle and late fruit maturity based on RNA-seq values in all three cultivars

(i) By comparing the RNA-seq data of flower buds during dormancy period, we identified 55 constitutive expressed genes from groups 1–5 while group 6 showed no expressions during the entire endodormancy cycle in ‘Suli’ pear (Additional file 4). We selected 19 differentially expressed genes mostly from subgroups 2A, 2C, 2D and 3D with period specific expressions during December and January when chilling accumulation and endodormancy release step proceed in ‘Suli’ pear as found in our previous study [35]. So to reconfirm their relations with bud endodormancy, the expression analysis was performed of all genes from subgroups 2D and 3D and randomly selected from subgroup 2A during flower bud endodormancy from October 15 to Feb 5 with 15 days interval in *P. pyrifolia* ‘Cuiguan’ (Fig. 5B). Expression results revealed that *PpNACs 37, 61, 70* (2A), *53, 151*(2D), *10, 92, 130* and *154* (3D) showed peaks in December and January and suggested that these *PpNAC* genes might be related to bud endodormancy (Fig. 5B-a, b, c) in ‘Cuiguan’ pear. Besides, stress related dehydrin genes which protect the cells from dehydration and desiccation damages by the environment during the dormancy process also showed high expressions in *P. pyrifolia* ‘Cuiguan’ during bud endodormancy period (Fig. 5B-d).
(ii) The transcriptomic data during color development in fruit of *P. pyrifolia* ‘Meirensu’ revealed 42 constitutive expressed *PpNAC* genes from groups 1–5 with some variations within groups and subgroups while there were no expressions in group 6 (Additional file 5). About 15 *PpNAC* genes showed period specific expressions after 1 day and 6 days (T7-T9 and T10-T12) under sunlight exposure and these genes were mostly from subgroups 1C, 2A and 4E (Fig. 6a). So, we randomly selected 2 and 3 genes for expression analysis from subgroups 2A (*PpNAC61, PpNAC70*) and 4E (*PpNAC172, PpNAC176, PpNAC23*) respectively for expression analysis. The qPCR results exposed that above NAC TFs, under blue light and dark treatments with 0 h, 12 h, 1 day, 3 days, 6 days and 10 days interval revealed some relations with anthocyanin accumulations in pear fruit as the kinetic of coloration and anthocyanin accumulation were already been recorded in our previous study [38]. The expressions of *PpNACs 61, 70, 172* and *176* were the highest after 1 day while sudden down after 3 and 6 days of blue light exposure as compared to dark conditions. In contrast, *PpNAC 23* showed low expressions at initial exposure days and high expressions with increasing exposure days of blue light than dark conditions (Fig. 6b). In conclusion, our results from RNA-seq and qPCR revealed that some genes of subgroups 2A and 4E might have some relation to fruit color developments in pear (Fig. 6).
(iii) We also examined the *PpNAC* genes from RPKM data during different fruit development stages in three pear species i.e. *P. pashia, P. calleryana*, and *P. pyrifolia* during 0, 7, 35 and 85 days after flowering to fruit maturity with two replicates of each (Fig. 7a, b, c). Heat map representations according to group and subgroup distributions demonstrated that subgroups 1E&4C, 1G&4E and 1F&5A played pivotal roles during early, middle and late fruit developments respectively in pear (Additional file 6). From the detailed evaluation, we found 22 differentially expressed genes that showed period specific expressions during fruit developments with minor differences among all species (Fig. 7a, b, c). Among them 5 *PpNACs* (1E, 2C, 4C and 4D) belonged to early, 7 (1G, 1D and 4E) associated to middle, 8 (1F and 5A) related to late fruit developments in pear while 2 (6A and 6B) showed higher expressions during fruit maturity in all species. Because we had no plant material during fruit developmental stages, so, from graphical representation based on RNA-seq values, we concluded that within differential expressed genes, *PpNAC 127* (1E), *PpNAC 46* (1G) and *PpNAC 56* (5A) were most probably related to early, middle and late fruit developments respectively in all pear species (Fig. 7d).

#### Expression profiles of *PpNAC* genes during stress tolerance pathways

**Fig. 8:**
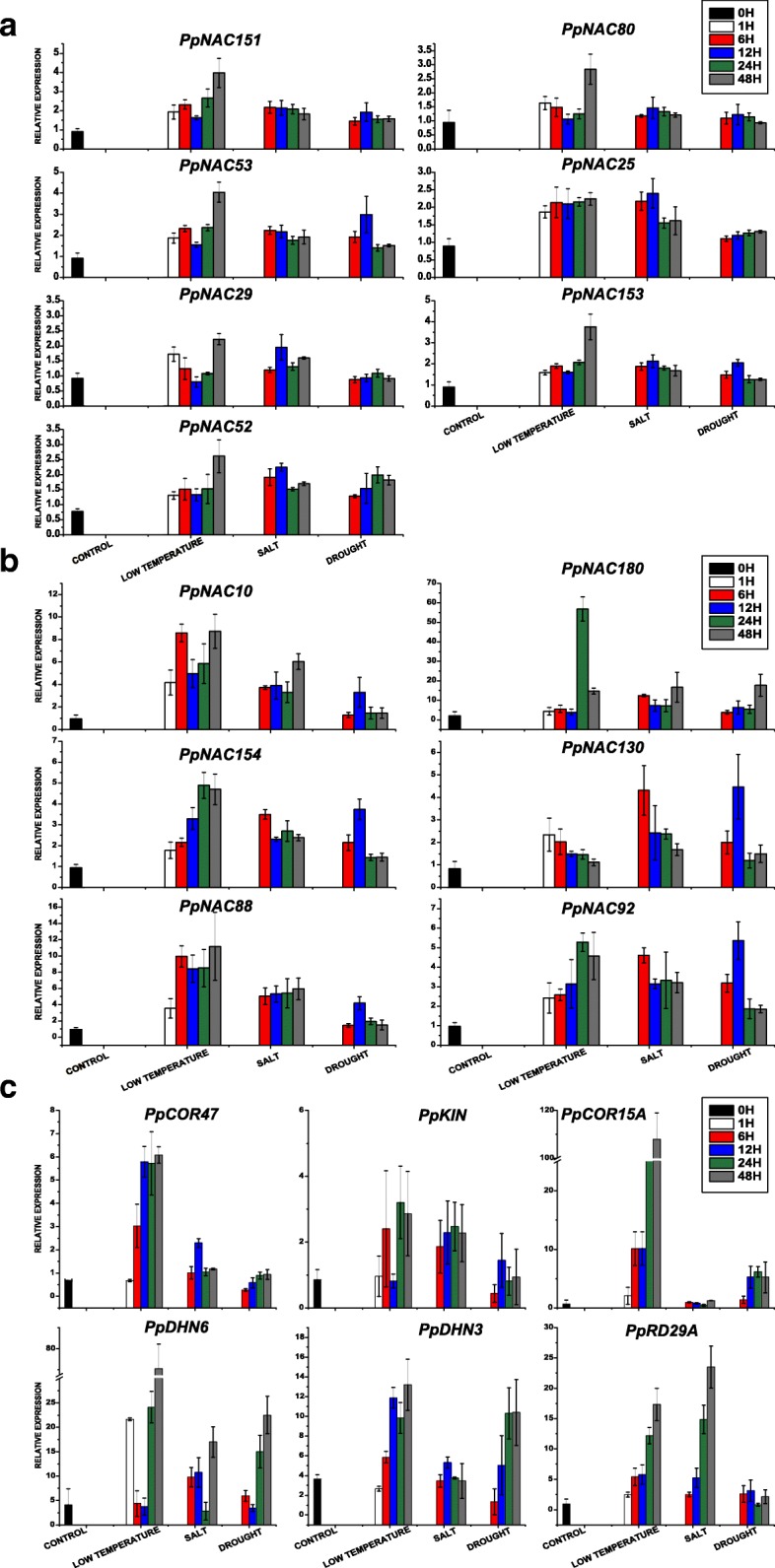
Expression evaluation of the identified *PpNAC* and stress related genes under different abiotic stresses in *P. pyrifolia* ‘Suli’. **a**, **b** qRT-PCR was used to examine the expression patterns of subgroups 2D and 3D in pear explants under low temperature (4 ^o^C), drought (15% PEG 6000) and high salinity (200 mM NaCl) stresses for 0 h, 1 h, 6 h, 12 h and 48 h represented by black, white, red, blue, green and gray bars in graphs respectively. **c**, To reconfirm the stress status and NAC relation during abiotic stresses, different stress responsive and *COR* genes are analyzed in same samples. The data were normalized to the pear *ACTIN* expression level and the mean expression value was premeditated from 4 independent replicates while the standard deviation are shown by vertical bars

Besides, NAC transcription factors had also been implicated in regulating the tolerances against different abiotic stresses in pear. Because we had no transcriptomic data of pear related to different abiotic stresses, so to find out the possible *PpNAC* genes related to different stresses, we passed through some membrane-associated NAC proteins including *NTLs* of Arabidopsis (*NTM1, NTM2, and NTL1–10*) and many stress-responsive marker genes (e.g., *ATAF1/2, ANAC019*) in subgroups 2D and 3D through smart blast (Table 1). Study of *MdNAC* genes in apple during stress conditions also helped us to reconfirm the subset of *MdNAC* genes (*MdNAC33*, *MdNAC143*, and *MdNAC168*) belonged to subgroup 3D and indicated a high level of sequence resemblances with the stress-related marker genes. These findings enforced us to check the subgroups 2D and 3D that they might have roles during stress conditions in pear. So, we started to give low temperature (4 °C), drought (15% PEG 6000) and high salinity (200 mM NaCl) stresses for 0 h, 1 h, 6 h, 12 h and 48 h to explants of *P. pyrifolia* ‘Suli’ to check the expressions of above subgroups. qPCR results demonstrated that all members of subgroups 2D and 3D had high expressions during low temperature, salt and drought stresses as compared to control (Fig. 8). In detail, *PpNACs 154&180*, *PpNACs 25&88*, *PpNACs 130&92* might have strong relations with freezing, salt and drought tolerances in pear plants respectively (Fig. 8a & b). To endorse the NAC relation and stress status of all explants, we checked the expressions of stress responsive genes in same samples and made a predicted decision that *PpCOR47&15A, PpRD29A&KIN* and *PpDHN3&6* might had relation with above stress related *PpNAC* genes during freezing, salt and drought tolerances respectively (Fig. 8c).

## Discussion

We found 185 *NAC* genes containing full ORFs in *Pyrus pyrifolia* White Pear Group through genome-wide analysis (Table 1). This TF seemed to be one of the largest compared with other plant species as per findings till now 117 *NAC* genes in Arabidopsis [3], 151 in Rice [28], 79 in grape [29], 152 in tobacco and soybean [30, 31] and 180 in Apple [39]. The largest numbers indicated that *NAC* genes had expanded with evolution [2, 9] in pear. It is relatively limited to use Arabidopsis NAC genes in phylogenetic tree as queries so to examine the evolutionary history, classification and functional prediction; we constructed a phylogenetic analysis of pear (185), apple (180) and Arabidopsis (138). Based on the full length sequences of NAC proteins, phylogenetic tree split the *PpNAC* genes into 6 distinct groups as previously found in Apple [32]. *PpNACs, MdNACs* and *AtNACs* genes from groups 1, 2, 3 and 5 indicated that these genes were not only homologous but might also be evolved from a common ancestor during evolution. In contrary, NACs from groups 4 and 6 implied that Rosaceae crops had common ancestors but different from Arabidopsis (Fig. 1).

Whole-genome duplication (WGD) event must had happened in pear about 30–45 MYA in prior to divergence with apple [34] and this gene duplications played vital roles in expansions, rearrangements and functional variations among NAC genes. So, collinerity of *PpNAC* genes and this WGD might have driven the expansions of this superfamily in pear and resulted in duplications of 44 *PpNAC* gene pairs. These duplications were observed in all groups but groups 4 and 6 showed only 2 and 1 pair of duplications respectively (Fig. 3). MEME showed that groups 1–5 had similar motifs (Motif 1-Motif 7) with minor changes and exchanges but in contrast, members of group 6 also showed some additional motifs (Motif 8, 9, and 10) (Fig. 4b). Gene structures also displayed no intronic regions in mostly members of group 6 (Fig. 4c). So, independent evolution, less duplication, different motifs and no intronic regions demonstrated that genes from groups 6 and 4 had functions of complicated transcriptional regulations only in woody perennial species and the members from Arabidopsis might have been lost in Rosaceae crops following the divergence during the evolutionary history. Our findings also demonstrated that genes in the same subgroups shared the similar gene structures and motifs and might have the similar gene functions (Fig. 4). Therefore, we did comparison with known functions of NAC genes in Arabidopsis and predicted the functions of candidate *PpNAC* genes according to best smart hit. As previous study reported that most of the subgroups in Arabidopsis were functioned in plant growth [40], signaling [7, 41] and stress response [42, 43] so, we also predict the same functions of these subgroups in pear except groups 4 and 6 (Table 1).

Gene expression is one of the basic evidence for gene functions. To find out the predicted functions of *PpNACs*, three different sets of RNA-Seq data provided us complete expression profiles of this superfamily during bud dormancy, fruit color and developments (Additional files 4, 5 and 6). Previously proved that *PbeNAC1* functioned in cold tolerance by interacting with bud dormancy and cold related *DREBs* and *DHN* genes [25, 44–46]. So, we found 9 *PpNAC* TFs (*PpNAC 37, 61, 70*, *53, 151*, *10, 92, 130* and *154*) and also 2 *DHNs* genes (*PpDHN3* and *PpDHN6*) that might be related to winter dormancy in pear (Fig. 5) because previously study on apple demonstrated that the reduction of free water content in dormant buds coincides with DHN protein accumulation during winter [39]. Regarding the role of NAC genes in anthocyanin accumulation and color development, *PpeNAC1* caused anthocyanin pigmentation in overexpressed transgenic tobacco [16], *AtNAC078* regulated the flavonoid biosynthesis under high-light in Arabidopsis [47] and in our previous study, an involvement of blue light was also found in anthocyanin accumulation in ‘Red Zaosu’ pear [36, 38]. Through RPKM data analysis and the homologous genes of *PpeNAC1* in pear, we found 15 *PpNAC* genes that might be related to light and anthocyanin accumulation (Fig. 6a) from which 5 *PpNAC* genes (*PpNACs 61, 70*, *172, 176* and *23*) possibly associated to blue light induced pigmentation in ‘Red Zaosu’ pear (Fig. 6b). Different fruit development stages in *P. calleryana*, *P. pashia* and *P. pyrifolia* revealed 22 differentially expressed genes mostly from groups 1 and 5 that might be related to fruit developments in pear (Fig. 6 and Additional file 4) while in apple 13 out of 182 *MdNAC* genes were found to be differentially expressed during the stages of fruit growth and maturity [48]. Similarly, predicted functions of groups 1 and 5 were also related to plant growth and development such as cell expansion [40], root initiation [49], vascular tissue development and secondary cell wall formation in Arabidopsis [50] (Table 1).

Due to the presence of stress-responsive marker genes in subgroups 2D and 3D, we found 13 *PpNAC* genes from these subgroups which were related to abiotic stresses in pear (Fig. 8) [32, 42] and previously 17 *MdNAC* genes were reported in apple that responded to one or more types of abiotic stresses [32]. Further, the expression levels of stress responsive genes (*NtRD29A, NtRD1, NtERD10D, NtLEA5, NtNCED1* etc) were higher in *PbeNAC1* transgenic tobacco plants as compared to WT under drought and low temperature stresses [25] which indicated that a transcription activator had an ability to activate a series of target genes that helped in adaptations and encountered these abiotic stresses [51, 52]. Likewise, in our study we also found highly expressed stress responsive genes *PpCOR47&15A, PpRD29A&KIN* and *PpDHN3&6* that might had relation with this transcription activator “*PpNAC*” during freezing, salt and drought tolerances.

NAC TFs have received much attention during different biological pathways of plant growth & developments and biotic & abiotic stresses [53, 54]. Accordingly, our findings indicated that *PpNAC* TFs not only played major roles during a number of biological processes of plant development like fruit development and maturity, anthocyanin accumulation and growth cessation but also during stress tolerance pathways like freezing, salt and drought in pear.

## Conclusions

Our work aimed to find the evolution, gene functions and expression patterns of NAC gene family in pear. Whole genome duplication (WGD) and duplication events played their important roles in expansion of this gene family in pear. Functional predications and expression divergences among different groups of *PpNAC* genes showed their involvement in multiple regulation pathways in pear. Multiple evidences supported that 9, 5 and 22 *PpNAC* genes belonged to bud endodormancy, fruit pigmentation and fruit developments respectively while 13 genes from subgroups 2D and 3D might be involved in responses to abiotic stresses in pear. Hence, these results of *PpNAC* TFs will help to build a solid foundation for the future exploration and potential improvements in the stress resistance, growth and development of pear as well as other crops through genetic engineering approaches.

## Methods

### Identifications of NAC TFs in pear

The predicted protein sequences to identify the members of NAC gene family were retrieved from the pear genome project (http://peargenome.njau.edu.cn/) [34]. First, a Hidden Markov Model search (HMM search) was done by using the pfam # PF01849 which contained *PpNAC* domains from domain analysis programs (http://pfam.xfam.org/). The other search was from Plant Transcription factor database (http://planttfdb.cbi.pku.edu.cn) of Pear NAC TFs. The sequences which were redundant, incomplete and high e-value had been rejected. Further, we used the SMART tool (http://smart.embl-heidelberg.de/) and the InterProScan tool (http://www.ebi.ac.uk/Tools/pfa/iprscan/) to find the presence of NAM domain in all selected NAC genes. The NAC TFs in Arabidopsis were downloaded from The Arabidopsis Information Resource (https://www.arabidopsis.org/) while for apple, NAC TFs were downloaded from GDR database (Genome Database for Rosaceae: http://www.rosaceae.org/) and Apple GFDB database (Apple Gene Function and Gene Family Database: http://apple.sdnx.co/) following the previous study on *MdNAC* TFs [32].

### Chromosome location and gene duplication of *PpNAC* genes

Chromosome locations of pear *NAC* genes were analyzed using BLAST software 2.25 (ftp:/ncbi.nlm.nih.gro/blast/executables/release/) to align the *NAC* sequences against the pear genome sequence and got the chromosome positions of all NAC genes [55]. The length of each chromosome was also obtained from pear genome database. The data were integrated and plotted by using Mapchart software (v.2.2) [56].

Collinearity blocks of whole genome and NAC TFs within the species were identified by using MCScanX algorithm with default settings and followed the given instructions on user manual with E-value ≤1e − 10 [57].

### Sequence alignment and phylogenetic analysis of *PpNAC* TFs

The sequences of pear, apple and Arabidopsis were aligned by using the MUSCLE program [58] while the phylogenetic trees for the *PpNAC, MdNAC* and *AtNAC* protein sequences were constructed by using the maximum likelihood method of the MEGA7.0 (Molecular Evolutionary Genetics Analysis) program [58]. The reliability of the trees was also tested using a bootstrapping method and the images of the phylogenetic trees were drawn.

### Gene structure and motif analyses

CDS sequences of each *PpNAC* gene with their corresponding full-length genomic sequences were uploaded on online website: Gene Structure Display Server (GSDS) (http://gsds.cbi.pku.edu.cn/) and the gene structures of the *PpNAC* gene family were obtained and downloaded. Moreover, conserved motifs were perceived in pear NAC family members using the motif analysis tool MEME (http://meme-suite.org/tools/meme) version 4.12.0 with the default parameters except for two: any number of repetitions; maximum number of motifs, 10.

### Plant material and RPKM data analysis

For stress treatments, vegetative buds of ‘Suli’ pear (*Pyrus pyrifolia* white pear group) were collected before bud break in March 2018. After bud sterilization and washing they were grown in 1/2 MS media and put in tissue culture room for getting pear seedlings. When the seedlings were uniform in size and at 6 to 8 leaves stage then they were randomly selected for stress treatments. For low temperature, seedlings were put at 4 °C while during drought and salt stresses, 15% PEG 6000 and 200 mM NaCl were added into 1/2 MS media respectively. Each sample with three replicates were collected after 0 h, 1 h, 6 h, 12 h and 48 h and immediately frozen in liquid nitrogen and RNA extracted without storage. The plant materials for coloration [38] and bud endodormancy [55] are the same as recently published.

Three different RNA-seq datasets i.e. pear bud endodormancy, fruit coloration and different fruit development stages were already been published [35–37]. MultiExperiment Viewer (v 4.8.1) software (www.tm4.org) was used to make the heat map of RNA-Seq data followed the given instructions on MeV quickstart guide.

### RNA extraction and cDNA synthesis

Total RNA was extracted from each sample using a modified cetyltrimethylammonium bromide (CTAB) method [59]. Genomic DNA was eliminated with DNase I and the concentration of total RNA was measured. First-strand cDNA was synthesized from 4 μg DNA-free RNA using the iScript cDNA Synthesis Kit (Bio-Rad, CA, USA) following the manufacturer’s instructions. The cDNA was diluted 10-fold and used as the template for real-time quantitative PCR (qRT-PCR) analysis.

### Real-time quantitative RT-PCR analysis

The solution of qRT-PCR reaction (15 μL total volume) was composed of 7.5 μL SYBR Premix Ex Taq™ (Tli RNaseH Plus), 1 μL of cDNA (corresponding to 0.5 μg total RNA), 0.5 μL from each forward and reverse primers (10 μM), and 5.5 μL RNase-free water. Following program: 30 s at 95 °C followed by 40 cycles of 95 °C for 5 s and 60 °C for 20 s was run for the reaction in a CFX Connect™ real-time PCR system (Bio-Rad, Hercules, CA, USA). Melting curves and sequencing of the qRT-PCR products confirmed the specificity of the qRT-PCR primers. Gene transcript levels were measured as 2^−ΔΔCt^ and normalized against *PpActin* (JN684184) transcript levels [35]. Data were subjected to one-way ANOVA while the Tukey’s range test (*P* < 0.05) was used to measure means among different treatments in all samples. Primer3 program (http://bioinfo.ut.ee/primer3-0.4.0/) is used to design the primers which are provided in Additional file 7.

## Additional files

Additional file 1: Detailed information, group wise distribution and protein sequences of selected pear NAC TFs. (XLSX 53 kb)

Additional file 2: Phylogenetic tree of apple, pear and Arabidopsis (DOCX 350 kb)

Additional file 3: Frequency Distribution of *PpNAC* genes on each chromosome. (DOCX 117 kb)

Additional file 4: Heat map presentation of all *PpNAC* TFs according to group distributions during bud endodormancy in *P. pyrifolia* Chinese ‘Suli’ (DOCX 144 kb)

Additional file 5: Heat map presentation of all *PpNAC* TFs according to group wise distributions during fruit color development in *P. pyrifolia* ‘Meirensu’ (DOCX 242 kb)

Additional file 6: Heat map presentation of all *PpNAC* TFs according to group distributions during fruit developments in three pear cultivars viz. *P. calleryana*, *P. pashia* and *P. pyrifolia.* (DOCX 925 kb)

Additional file 7: All primer sequences used for qRT-PCR analysis (DOCX 138 kb)

## Abbreviations

BLAST: The Basic Local Alignment Search Tool
HMM: Hidden Markov Model
MEGA: Molecular Evolutionary Genetics Analysis
MEME: Multiple Em for Motif Elicitation
MUSCEL: Multiple Sequence Comparison by Log-Expectation
ORF: Open reading frame
Pfam: Protein family
RNA-seq: Ribose nucleic acid sequencing
RPKM: Reads per kilobase per million reads
SMART: A Simple Modular Architecture Research Tool
TF: Transcription factor
