# Supplementary material for: Genome wide identification and predicted functional analyses of NAC transcription factors in Asian pears
Source: BMC Plant Biol. 2018 Oct 3;18:214. doi: 10.1186/s12870-018-1427-x (PMC6169067; doi:10.1186/s12870-018-1427-x)
Supplement: Supplementary file 1 [file 12870_2018_1427_MOESM2_ESM.docx]

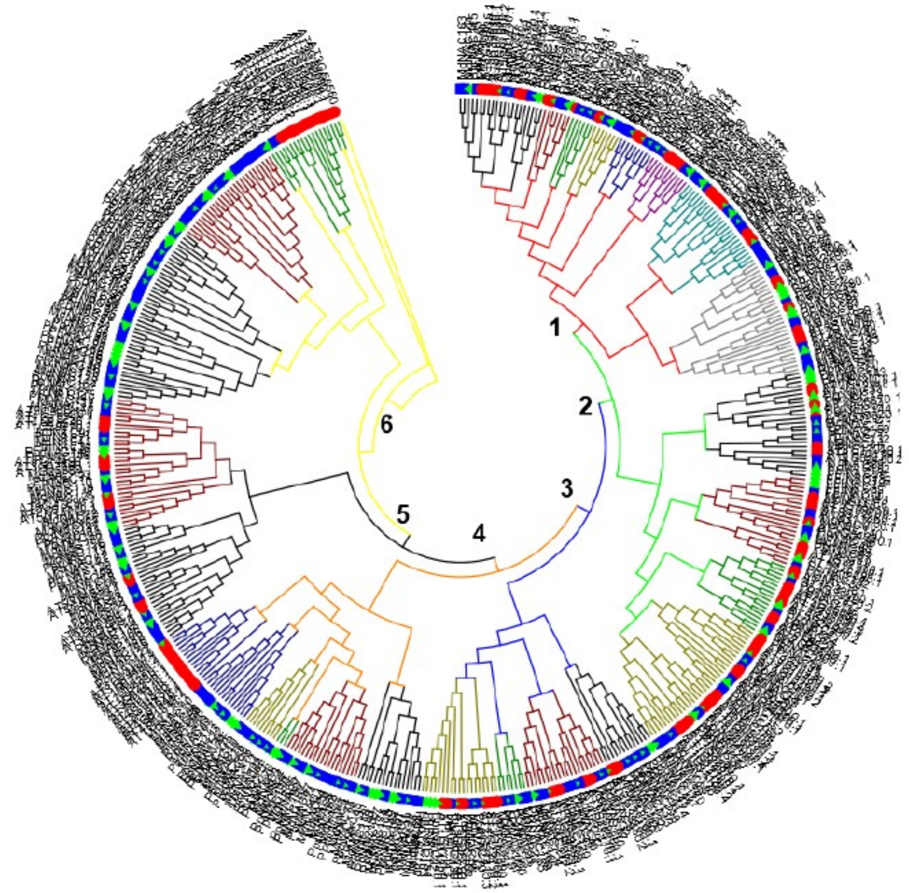

Additional file 2: Phylogenetic analysis of the NAC transcription factors of Arabidopsis, apple and pear represented by red cricles, blue squares and green triangles respectively with annotations. The tree was divided into 6 main groups 1, 2, 3, 4, 5 and 6 represented by red, green, blue, orange, black and yellow colored clusters within the tree with their subgroups designated from 1A-1H, 2A-2D, 3A-3D, 4A-4E, 5A-5B and 6A-6C respectively outside the tree.
