# Supplementary material for: Genome wide identification and predicted functional analyses of NAC transcription factors in Asian pears
Source: BMC Plant Biol. 2018 Oct 3;18:214. doi: 10.1186/s12870-018-1427-x (PMC6169067; doi:10.1186/s12870-018-1427-x)
Supplement: Supplementary file 2 [file 12870_2018_1427_MOESM3_ESM.docx]

Additional file 3: Frequency Distribution of PpNAC genes on each Chromosome according to group distributions

Chr #	*PpNAC* Genes		Group 1		Group 2		Group 3		Group 4		Group5		Group 6	
	Gene#	Frequency	Genes#	Frequency	Gene#	Frequency	Gene#	Frequency	Gene#	Frequency	Gene#	Frequency	Gene#	Frequency
Chr#1	4	2.1	3	7.5	1	2.9	0	0	0	0	0	0	0	0
Chr#3	14	7.5	1	2.5	0	0	3	13.0	1	3.3	3	12.5	6	17.6
Chr#4	8	4.3	1	2.5	1	2.9	1	4.3	4	13.3	2	8.3	1	2.9
Chr#5	14	7.5	5	12.5	5	14.7	1	4.3	0	0	0	0	1	2.9
Chr#6	11	5.9	4	10	2	5.8	2	8.6	1	3.3	0	0	2	5.8
Chr#7	3	1.6	0	0	3	8.8	0	0	0	0	0	0	0	0
Chr#8	4	2.1	1	2.5	1	2.9	0	0	0	0	2	8.3	0	0
Chr#9	8	4.3	1	2.5	4	11.7	0	0	0	0	2	8.3	1	2.9
Chr#10	17	9.1	7	17.5	6	17.6	0	0	3	10	1	4.1	0	0
Chr#11	21	11.3	1	2.5	2	5.8	4	17.3	1	3.3	0	0	13	38.2
Chr#12	5	2.7	1	2.5	0	0	1	4.3	1	3.3	2	8.3	0	0
Chr#13	5	2.7	0	0	1	2.9	1	4.3	0	0	2	8.3	1	2.9
Chr#14	9	4.85	4	10	0	0	2	8.6	2	6.6	1	4.1	0	0
Chr#15	14	7.5	5	12.5	2	5.8	1	4.3	0	0	4	16.6	2	5.8
Chr#16	2	1.0	2	5	0	0	0	0	0	0	0	0	0	0
Chr#17	9	4.8	1	2.5	3	8.8	0	0	0	0	3	12.5	2	5.8
Cun1	21	11.3	1	2.5	2	5.8	3	13.0	10	33.3	1	4.1	4	11.7
Cun2	16	8.6	2	5	1	2.9	4	17.3	7	23.3	1	4.1	1	2.9
**Total**	**185**	**100**	**40**	**100**	**34**	**100**	**23**	**100**	**30**	**100**	**24**	**100**	**34**	**100**
