# Supplementary material for: Genome wide identification and predicted functional analyses of NAC transcription factors in Asian pears
Source: BMC Plant Biol. 2018 Oct 3;18:214. doi: 10.1186/s12870-018-1427-x (PMC6169067; doi:10.1186/s12870-018-1427-x)
Supplement: Supplementary file 3 [file 12870_2018_1427_MOESM4_ESM.docx]

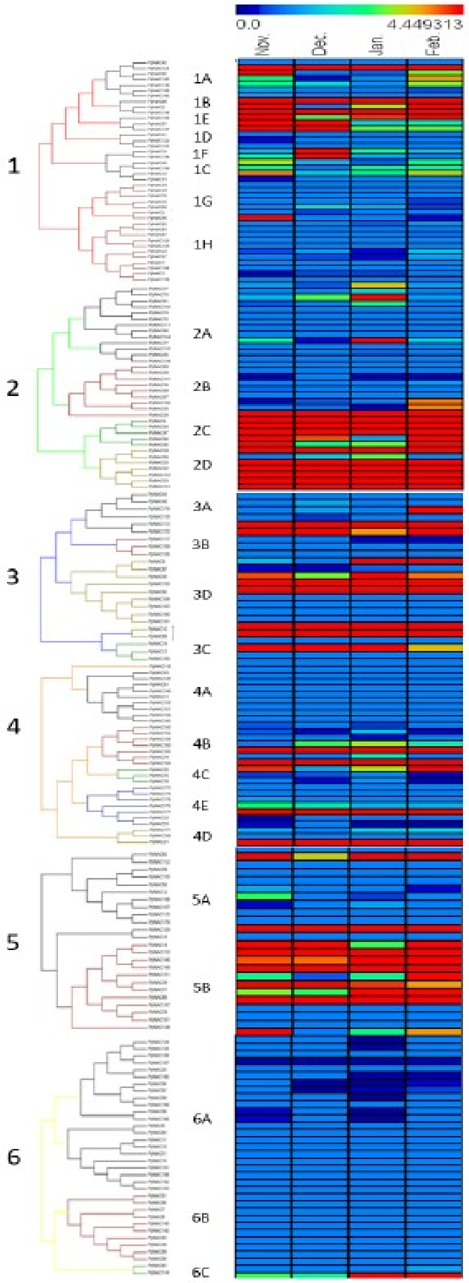

Additional file 4: Heat map presentation of *PpNAC* TFs according to group and subgroup distributions during bud endodormancy in *Pyrus pyrifolia* cv. Chinese white pear groups from November to February (entire dormancy period). The rainbow color scales present at the top of heat map represents log2 values of RPKM data.
