# Supplementary material for: Genome wide identification and predicted functional analyses of NAC transcription factors in Asian pears
Source: BMC Plant Biol. 2018 Oct 3;18:214. doi: 10.1186/s12870-018-1427-x (PMC6169067; doi:10.1186/s12870-018-1427-x)
Supplement: Supplementary file 4 [file 12870_2018_1427_MOESM5_ESM.docx]

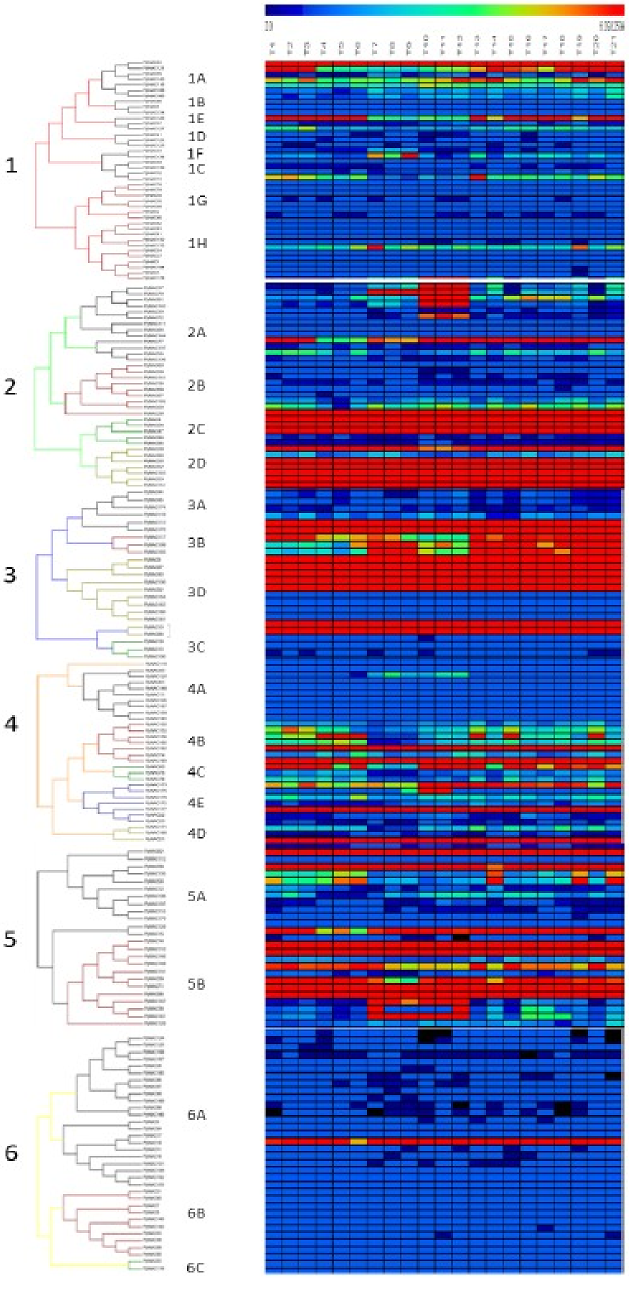


**Additional file 5:** Expression representation of *PpNAC* TFs during fruit color development under sun light and dark light treatments i.e. T1-T3, T4-T6, T7-T9 and T10-T12 indicates 0hour, 6hours, 1day and 6days under sun light conditions while T13-T15, T16-T18 and T19 to T21 represents 6hours, 1 day and 6 days treatments under dark conditions respectively *in Pyrus pyrifolia* cv. Meirensu. The rainbow color scales present at the top of heat map represents log2 values of RPKM data.
