# Supplementary material for: Genome wide identification and predicted functional analyses of NAC transcription factors in Asian pears
Source: BMC Plant Biol. 2018 Oct 3;18:214. doi: 10.1186/s12870-018-1427-x (PMC6169067; doi:10.1186/s12870-018-1427-x)
Supplement: Supplementary file 5 [file 12870_2018_1427_MOESM6_ESM.docx]

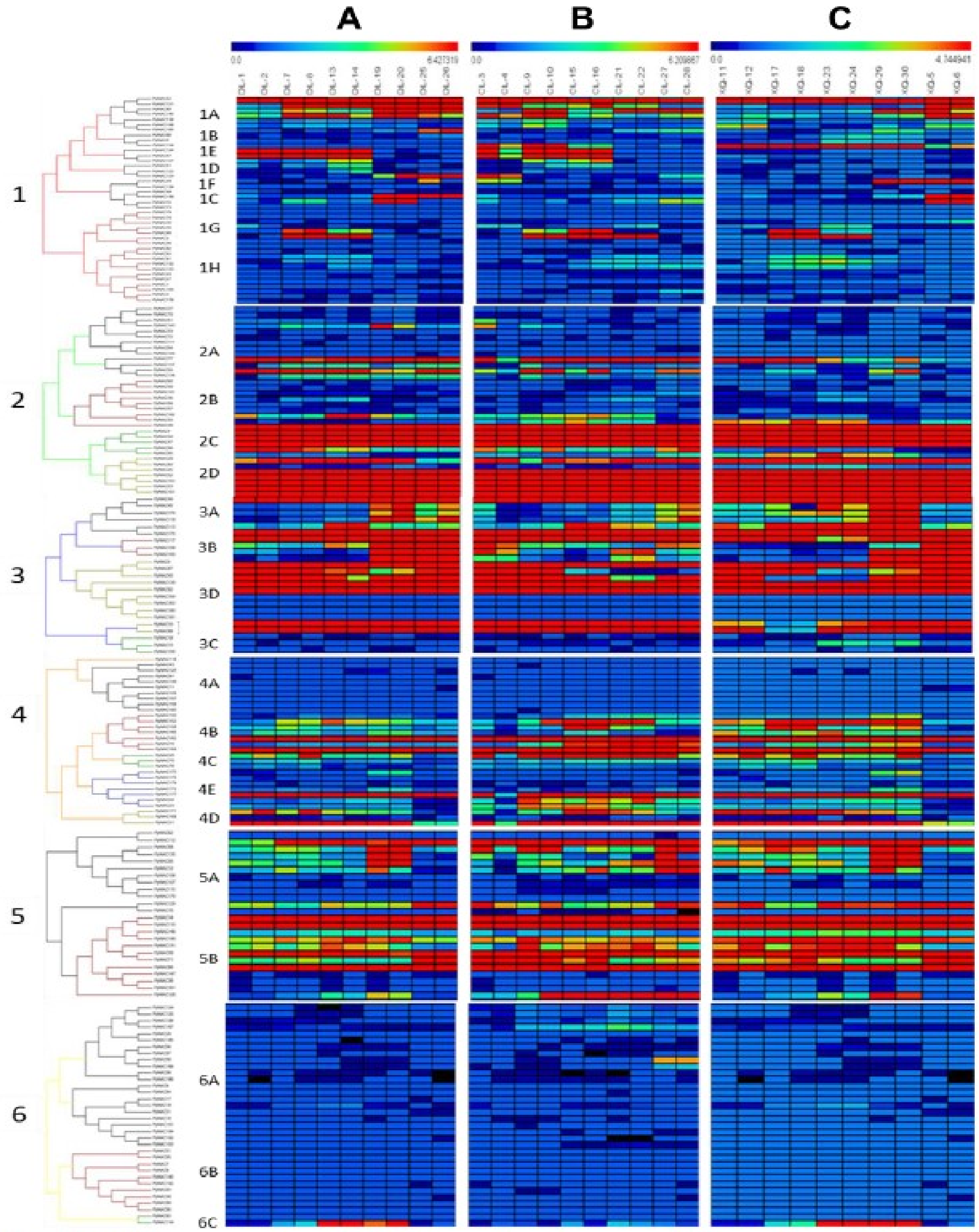

Additional file 6: Heat map presentation of *PpNAC* TFs according to their groups and subgroups during fruit development in three different pear cultivars. A,B,C Expression profiles of *PpNAC* TFs from 0, 7, 35 and 85 days after flowering to fruit maturity with two replicates in *Pyrus calleryana, Pyrus pashia and Pyrus pyrifolia* respectively. The rainbow color scales present at the top of heat map represents log2 values of RPKM data.
