# Supplementary material for: Genome wide identification and predicted functional analyses of NAC transcription factors in Asian pears
Source: BMC Plant Biol. 2018 Oct 3;18:214. doi: 10.1186/s12870-018-1427-x (PMC6169067; doi:10.1186/s12870-018-1427-x)
Supplement: Supplementary file 6 [file 12870_2018_1427_MOESM7_ESM.docx]

Additional file 7: All primer sequences used for qRT-PCR analysis

Purpose	Gene Name	Forward Primer (5' to 3')	Reverse Primer (5' to 3')
Stress Related *PpNAC* Genes (Subgroup 2D and 3D)	*PpNAC25*	AAACACACCTGGCCCATATC	CCATGGGAAGCATGAGACTT
	PpNAC28	TCACGGTCTGTTGGATTGAA	CCATATTGCTCGCCATTTTT
	PpNAC29	AAGGAGACCGAACAGACTGG	GTCACTCGGCAGAACAAGGT
	PpNAC52	GGGATGAAGAAGACGCTGGT	CCTCTTCCCCAGGTACACAA
	PpNAC53	CACCCAACAGACGAGGAACT	GCCTTCCAGTACCCTTGGTT
	PpNAC80	AAGGAGAGCGAACAGACTGG	GTTACTCGGCAGCACAAGGT
	PpNAC151	ACTATCCGTCACCAGGTCCA	CTCCACCAGTGCAGACAGAA
	PpNAC153	CGTGACTGGTGTACCTGGTG	TTCCCCAGTGCCTATCACAG
	PpNAC10	GCTCTTCTCAGCTCGACGAC	GGTTGAAGGGAGACACGTCA
	PpNAC88	ACGAGGAGCTTCTGGTTCAG	GTTCCAGTGGCCTTCCAGTA
	PpNAC92	GTACGGGGAAAAGGAGTGGT	GCCTGAGGCTGTTCTTCTTC
	PpNAC130	GACGACGACGGACTACTCAAG	GCGTGGAGGTAGGAGAACAT
	PpNAC154	TCCACTTAACGGACGAGGAG	GCCTTCCAGTAACCGCTTC
	PpNAC180	GTCCGTCGATTTCCAGCTAC	GCCTTCCAGTAACCGCTTC
Dormancy and color related *PpNAC* genes	*PpNAC23*	TGTCGCACAAGAGTCACCAA	TTCCCCTATGATTGCGGTGG
	PpNAC176	GAGCTTTCACCCATCGACTC	TAACCCAGTCGGTCTTTTGG
	PpNAC172	GTACCCAAAAACCGGTCAAG	TGCAGCATCCACTTTCTCAC
	PpNAC61	TGAGTTTCGCCTTCCTCCTA	GGTTTTGTCTGGTCGTGGAT
	PpNAC70	GCCGGATTATGAAGCGCAAT	CAGCTGGTCTCGGAAGAACG
	PpNAC37	TGCCGGATTTTGAAGCGAGA	GCCAGCCTAGGGAGAATTGG
	PpNAC143	GTAAGACCCCTCAAGGCTCC	TAGTGCCTTTGCCAGCACTC
Stress Responsive Genes	*PpDHN3*	CCGAGGAAGAACACAAGGACA	AGCCACAAATGACACCACCA
	PpDHN6	CGCACCATGAAAAGAAGGGC	ACGCCAAGCACCGGTTATTA
	PpCOR47	GGTTGTCCATCACCACGAAG	ATGATGGTCGTGACTCTGCT
	PpCOR15A	GGAAACCCAATTCAACATGG	TCCATCATCCCTCTTTCCTG
	PpKIN	AACCAATCCCAGAACATGAGC	AGCCTTCTCCATCATTTGCTG
	PpRD29A	CACGTCTGCCATTGTTGACA	AACTGCGGCTGTAATACCCT
Actin	*PpActin*	CCATCCAGGCTGTTCTCTC	GCAAGGTCCAGACGAAGG
